# Label-Free Assessment of Mannitol Accumulation Following Osmotic Blood–Brain Barrier Opening Using Chemical Exchange Saturation Transfer Magnetic Resonance Imaging

**DOI:** 10.3390/pharmaceutics14112529

**Published:** 2022-11-20

**Authors:** Jing Liu, Chengyan Chu, Jia Zhang, Chongxue Bie, Lin Chen, Safiya Aafreen, Jiadi Xu, David O. Kamson, Peter C. M. van Zijl, Piotr Walczak, Miroslaw Janowski, Guanshu Liu

**Affiliations:** 1 Department of Radiology, The First Affiliated Hospital of Guangzhou Medical University, Guangzhou 510230, China; 2 Russell H. Morgan Department of Radiology and Radiological Sciences, Division of MR Research, The Johns Hopkins University School of Medicine, Baltimore, MD 21205, USA; 3 F.M. Kirby Research Center for Functional Brain Imaging, Kennedy Krieger Institute, Baltimore, MD 21205, USA; 4 Department of Diagnostic Radiology and Nuclear Medicine, University of Maryland, Baltimore, MD 21201, USA; 5 Department of Biomedical Engineering, Johns Hopkins University, Baltimore, MD 21218, USA; 6 The Sidney Kimmel Comprehensive Cancer Center, Johns Hopkins University, Baltimore, MD 21218, USA; 7 Department of Neurology, Johns Hopkins University, Baltimore, MD 21218, USA

**Keywords:** CEST MRI, MRS, mannitol, BBBO treatment

## Abstract

Purpose: Mannitol is a hyperosmolar agent for reducing intracranial pressure and inducing osmotic blood–brain barrier opening (OBBBO). There is a great clinical need for a non-invasive method to optimize the safety of mannitol dosing. The aim of this study was to develop a label-free Chemical Exchange Saturation Transfer (CEST)-based MRI approach for detecting intracranial accumulation of mannitol following OBBBO. Methods: In vitro MRI was conducted to measure the CEST properties of D-mannitol of different concentrations and pH. In vivo MRI and MRS measurements were conducted on Sprague-Dawley rats using a Biospec 11.7T horizontal MRI scanner. Rats were catheterized at the internal carotid artery (ICA) and randomly grouped to receive either 1 mL or 3 mL D-mannitol. CEST MR images were acquired before and at 20 min after the infusion. Results: In vitro MRI showed that mannitol has a strong, broad CEST contrast at around 0.8 ppm with a mM CEST MRI detectability. In vivo studies showed that CEST MRI could effectively detect mannitol in the brain. The low dose mannitol treatment led to OBBBO but no significant mannitol accumulation, whereas the high dose regimen resulted in both OBBBO and mannitol accumulation. The CEST MRI findings were consistent with ^1^H-MRS and Gd-enhanced MRI assessments. Conclusion: We demonstrated that CEST MRI can be used for non-invasive, label-free detection of mannitol accumulation in the brain following BBBO treatment. This method may be useful as a rapid imaging tool to optimize the dosing of mannitol-based OBBBO and improve its safety and efficacy.

## 1. Introduction

The blood-brain barrier (BBB) poses a major challenge in drug delivery into the central nervous system. For example, the fact that less than 2% of FDA approved anticancer drugs can pass the BBB is thought to be a major factor in the in the low success rate in brain cancer clinical trials [1,2]. Thus, there has been a recently renewed clinical interest in BBB opening methods to overcome these challenges. Mannitol is a sugar alcohol that has been used widely in the clinic as an intravenous (IV) hyperosmotic diuretic agent used for lowering intracranial and intraocular pressure (ICP) complicating traumatic brain injury [3], stroke [4], brain tumors [5], and other conditions [6,7]. Intra-arterial (IA) infusion of mannitol results in local osmotic blood–brain barrier opening (OBBBO) in the cerebral vascular territory supplied by the catheter [8,9]. Since the first Phase I clinical trial in 1979 [10], many clinical studies have shown that, combined with OBBBO, intra-arterial (IA) drug infusion can effectively improve CNS delivery of chemotherapy drugs [11]. For example, Charest et al., showed that combined IA-drug infusion and mannitol-based OBBBO generated an overall 2-fold higher carboplatin concentrations in brain tumors compared to IA infusion alone [12]. Thus, for agents that cannot cross the BBB naturally, such as antibodies or nanobodies, there is a great clinical demand for the development of quantitative imaging biomarkers that could provide spatial information about the extent of BBB opening and drug delivery [13,14].

While numerous new strategies to deliver drugs beyond the BBB have been explored to date, such as microbubble-based high-intensity focused ultrasound (HIFU) and convection-enhanced delivery (CED), or receptor-mediated transcytosis (RMT), mannitol-based OBBBO is still considered an effective and safe approach that is continuously used in the clinic [15]. However, the current mainstay of X-ray guidance of OBBBO procedures lacks spatial selectivity and may not detect off-target OBBBO and possible unwanted adverse effects in normal brain parenchyma. To overcome this limitation, contrast-based MRI methods are being developed to pre-determine the regions affected by IA-injected mannitol and to provide image-guidance for fine-tuning key injection parameters to achieve precisely controlled OBBBO in desired areas [16,17,18,19]. Real-time MRI has been also used to navigate OBBBO in a patient with brain cancer [20,21].

A critical hindrance to the widespread use of IA-injection of mannitol in the brain is the potential of mannitol deposition following OBBBO. It is well known that the accumulation of mannitol in the brain interstitium can cause a “rebound” phenomenon, i.e., a secondary reversal of the osmotic gradient and an increase in brain water content, followed by increased ICP [22,23]. This can be caused by either prolonged or repeated dosing [24,25]. However, in infiltrative brain tumors, interstitial mannitol concentrations many folds higher than in the plasma can be observed even after a single intravenous bolus injection [26]. Thus, the dose and frequency of mannitol administration need to be carefully planned and monitored to avoid this potential side effect while maintaining treatment efficacy. Clinically, either serum osmolarity or osmolar gap are used as surrogate measures of serum mannitol concentration [7]. The osmolality is directly measured by the concentration of solutes per kilogram of solvent (mOsm/kg), and the osmotic (osmole, or osmolal) gap is calculated by the difference between osmolality and osmolarity, the calculated value of the concentration of solutes per liter of solvent (mOsm/L). An osmolality of 320 mOsm/kg and an osmolar gap of 20–55 mOsm/kg were suggested to be the highest safe serum mannitol level [27]. However, neither of these surrogate measures reflect absolute values of serum mannitol, making it difficult for clinicians to closely monitor intravascular volume status and renal function during mannitol therapy. A few previous studies reported the use of ^1^H magnetic resonance spectroscopy (MRS) and magnetic resonance spectroscopic imaging (MRSI) for detecting mannitol [28,29,30]. However, a low spatial resolution and a relatively long acquisition time hamper the clinical application of MRS/MRSI. Therefore, a new non-invasive method that can reliably and rapidly assess the amount and spatial distribution of mannitol accumulation in the brain is needed.

This study aimed to develop an MRI approach for detecting mannitol accumulated in the brain following OBBBO. The mannitol molecule contains six hydroxyl(-OH) protons, which can be directly detected by Chemical Exchange Saturation Transfer (CEST) without the need for chemical labeling [31]. CEST MRI is an emerging technology wherein the MRI contrast is generated by transferring the selectively saturated MR signal of exchangeable protons to their surrounding water protons through the process of chemical exchange [32]. Mounting studies have shown the ability of CEST MRI to detect hydroxyl-protons containing molecules such as glucose [33,34,35], 2-deoxy-D-glucose (2-DG) [36,37,38], 3-O-methyl-D-glucose (3OMG) [39,40], glucosamine [41], dextrans [42,43,44,45], and many others [46,47]. Some of these methods have entered patient studies [48,49]. Herein, we investigated the ability of CEST MRI to detect the tissue accumulation of mannitol at a high spatial resolution, which will provide a valuable imaging tool for optimizing mannitol-based BBBO treatments by controlling the dose of mannitol to prevent unintended mannitol leakage to the brain parenchyma.

## 2. Materials and Methods

### 2.1. Chemicals

Unless otherwise noted, all chemicals were purchased from Sigma Aldrich (St. Louis, MO, USA). The D-mannitol (25% solution) used in animal studies was purchased from Hospira Inc. (Hospira Inc., Lake Forest, IL, USA).

### 2.2. Animals

All animal protocols were approved by our Institutional Animal Care and Use Committee. Sprague-Dawley rats (male, 200–250 g) were purchased from Charles River (Watershed, MA, USA). Catheterization was performed as previously described [16,50]. In brief, rats were anesthetized under 2% isoflurane and fixed in the supine position, followed by dissection of the common carotid artery (CCA) bifurcation. The external carotid artery (ECA) and pterygopalatine artery (PPA) were temporarily ligated with 4–0 silk sutures to permit all the solutions to enter the cerebrum. A small arteriotomy in the CCA was made, and a catheter (VAH-PU-C20, Instech Solomon Inc., Plymouth Meeting, PA USA) connected to #30 PTFE tubing was introduced into the internal carotid artery (ICA). The intra-arterial catheter was secured with the animal during MRI scans. Rats were randomly separated into two groups (*n* = 3 each) to receive either 1 mL or 3 mL D-mannitol at a 0.6 mL/min rate. The corresponding infusion time and total doses were 1.7 min (4 mL/kg or 1 g/kg) and 5 min (12 mL/kg or 3 g/kg), respectively.

### 2.3. ^1^H-NMR

The ^1^H-NMR measurements of mannitol solutions (100 mM in D_2_O or a mixture of 90% H_2_O and 10% D_2_O) were performed on a Bruker Avance III 500 MHz spectrometer (Bruker Biosciences, Billerica, MA, USA) at room temperature (pH = 6.5). The ^1^H NMR spectra were acquired using a standard one-dimensional pulse sequence (zg30) with 128 scans. A total of 65,500 data points for each scan were acquired with a spectral width of 20.6 ppm and an acquisition time of 3.18 s. The data were recorded and processed with TopSpin 4.0.1.

### 2.4. In Vitro MRI

In vitro MRI was conducted to measure the CEST properties and T_1_ and T_2_ relaxation times of D-mannitol in PBS solution of different concentrations (5 to 100 mM) and pH (5.5 to 8.0) using a 11.7T Bruker Avance vertical bore MRI system equipped with a 15 mm sawtooth RF coil (Bruker Biosciences, Billerica, MA, USA). CEST MRI was conducted using a modified rapid acquisition with relaxation enhancement (RARE) sequence as described previously [44,51]. Z-spectral data were acquired by varying the offset of saturation pulses from −4 ppm to 4 ppm with respect to the water resonance (0 ppm) using a increment of 0.2 ppm, with the S_0_ image was acquired using B_1_ = 0 µT at −50 ppm. Unless otherwise noted, the parameters of the saturation RF pulses were continuous wave (CW), T_sat_ = 3000 ms, B_1_ = 1.8 μT (150 Hz). Other imaging parameters were: TR/TE = 6.0 s/5 ms, RARE factor = 16, slice thickness = 2 mm, matrix size = 64 × 64, FOV = 15 × 15 mm^2^, spatial resolution= 0.23 × 0.23 mm^2^, number of averages (NA) = 2, total acquisition time = 16 min 48 s. The B_0_ inhomogeneity was measured using the Water Saturation Shift Reference (WASSR) method [52] using the same parameters as those used in CEST imaging except TR = 1.5 s, T_sat_ = 500 ms, B_1_ = 0.5 μT (21.3 Hz) and the saturation frequency swept from −1 ppm to 1 ppm (step size = 0.1 ppm). T_1_ and T_2_ maps were acquired as described previously [16,53] using a RARE-based saturation recovery sequence with eight TR values ranging between 200 ms and 15,000 ms (TE = 4.3 ms and RARE factor = 4, central encoding), and a modified RARE pulse sequence (TR/TE = 25,000/4.3 ms and RARE factor = 16) with a Carr-Purcell-Meiboom-Gill (CPMG) T_2_ preparation module (t_CPMG_ = 10 ms, the number of CPMG loops ranging from 2 to 1024, corresponding to echo times = 20 ms to 10.24 s), respectively. During MRI scans, the temperature of the samples was maintained at 37 °C.

### 2.5. In Vivo MRI

All in vivo studies were conducted on a Biospec 11.7T horizontal MRI scanner (Bruker Biosciences, Billerica, MA, USA) equipped with a rat brain surface array RF coil (receiver) and a 72 mm volume coil (T11232V3, transmitter). All CEST MR images were acquired using the same RARE-CEST sequence (CW saturation pulse: B_1_ = 1.8 µT, T_sat_ = 3 s, TR/TE = 5000/5 ms, RARE factor = 23, matrix size = 64 × 64 (with partial FT acceleration to 64 × 23), FOV = 30 × 30 mm^2^, spatial resolution = 0.47 × 0.47 mm^2^, slice thickness = 0.6 mm, number of averages NA = 2, acquisition time = 8 m 40 s). Z-spectral acquisitions were acquired by sweeping saturation offsets (CW pulse, B_1_ = 1.8 µT, T_sat_ = 3 s) from −5 to +5 ppm (step=0.2 ppm) before and at 20 min after the infusion of mannitol at the prescribed volumes. The time interval of 20 min was chosen because the half-life of mannitol in rats was estimated to be 17–25 min using the reported human values of 70–100 min [54,55] and a scaling method reported by Obach and colleagues [56]. The WASSR method was also acquired to correct B_0_ inhomogeneity.

T_1_ and T_2_ maps were acquired using the same geometry as the CEST MRI. In brief, T_1_ maps were assessed using a RARE-based saturation recovery sequence with six TR values ranging between 375 ms and 7,500 ms; TE = 11.3 ms and RARE factor = 4, central encoding, acquisition time = 3 m 47 s. T_2_ maps were acquired using a multi-slice/multi-echo (MSME) sequence (TR = 2200, 30 TE values ranging from 7.5 to 225 ms, acquisition time = 2 m 21 s).

Following the post-mannitol CEST acquisition, single-voxel localized ^1^H MRS spectra (voxel size = 4 × 6 × 3 mm^3^) were acquired in the ipsilateral and contralateral hemispheres using a stimulated echo acquisition mode (STEAM) sequence (TE = 4 ms, TM = 10 ms, TR = 2.5 s, NA = 64, acquisition time = 2 m 40 s) with outer volume suppression (OVS) and the variable power RF pulses and optimized relaxation delays (VAPOR) water suppression scheme according to previously published procedure [57]. A field mapping method was used to adjust the first-and second-order shims before MRS acquisition.

### 2.6. Data Processing

All MRI data processing was performed using custom-written scripts in MATLAB (Mathworks, Waltham, MA, USA). For CEST MRI data, pixel-wise B_0_ correction was conducted first. Then a region of interest (ROI) mask was placed over each sample, and the mean value of water signal intensity (S^Δω^) at each saturation offset relative to the water protons (±Δω) was calculated. The normalized S^Δω^/S_0_, where S_0_ is the water signal intensity without saturation, was plotted as a function of Δω, which is traditionally called a Z-spectrum. To quantify the CEST effect, the magnetization transfer ratio asymmetry (MTR_asym_) was calculated as MTR_asym_ = (S^−∆ω^ − S^+∆ω^)/S_0_. As the peak position of MTR_asym_ plots is dependent of the concentration of mannitol due to interference of the direct saturation, we used numerically integrated area under the curve (AUC) [58] of MTR_asym_ plots between 0.2 and 2 ppm as an approximation of the signal integral. Because AUC values are also dependent on the frequency range, we normalized the AUC values by the spectral width (ppm) that used in the calculation. The resulting AUC values have a unit of % per ppm (%/ppm).

To accurately quantify the CEST effect in the presence of substantial T_1_ changes, the T_1_ compensated inverse Z-analysis (AREX), defined by (S_0_/S^+∆ω^ − S_0_/S^−∆ω^)/T_1_, were also calculated using the acquired T_1_ times [59,60,61]. Of note, because the AREX values were calculated to quantify the asymmetry between the water signals at two offsets with respect to water resonance, it actually should be called AREX_asym_.

To quantify the concentration of mannitol in different tissues, we also fitted the Z-spectra using 6-pool Bloch equations as described previously [62,63,64]. To reduce the computing time and increase the reliability of fitting, we performed Bloch fitting in two steps. First, we fitted using the ROI values of ipsilateral and contralateral hemispheres, which had higher SNR compared to those of individual pixels. In the fitting, water R_1A_ (experimentally measured), the offsets of amide, amine, and magnetization transfer contrast (MTC) pools, and the T_2_ times of all the exchangeable protons (except the NOE pool) were fixed, with all other parameters set to fitting parameters. Table 1 lists the starting or fixed values according to those reported in the literature [65]. Then, the fitted exchange rates of all pools, T_2_ relaxation time of the NOE pool, and concentrations of amide and NOE from the first step ROI fitting were fixed in the second step pixel-by-pixel fitting.

In vivo ^1^H-MRS was processed according to a previously published procedure [66]. As a reference for chemical shifts, the *N*-acetyl-aspartate (NAA) resonance was set at 2.02 ppm. After local baseline flattening around the integration region using a second-order polynomial baseline correction, the peaks at 3.03 ppm and 3.5–4 ppm (total creatine (tCr) and mannitol, respectively), were quantified using their integrated peak areas.

### 2.7. Statistical Analysis

Data were expressed as mean ± SD. The comparison of two groups was conducted using a two-tailed Student’s t-test.

## 3. Results

### 3.1. CEST Characteristics of mannitol

**Figure 1:**
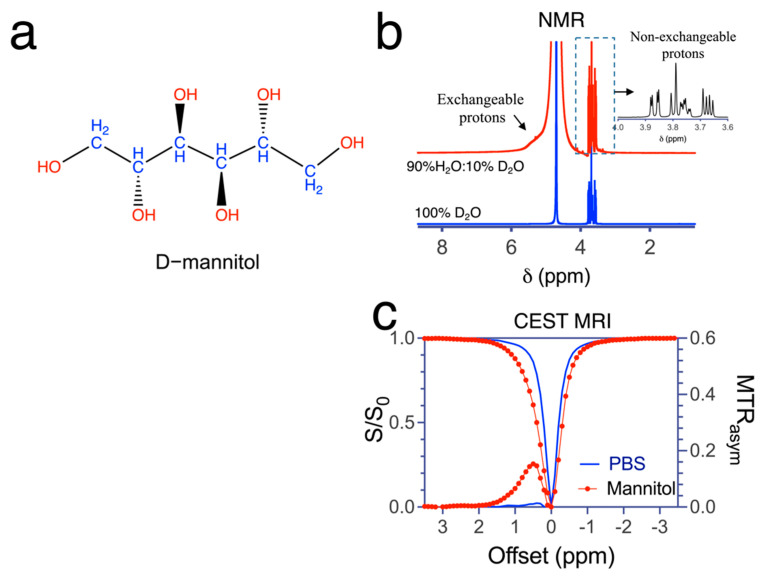
NMR and CEST MRI signal of mannitol (**a**) Chemical structure of mannitol. Red: functional groups containing exchangeable protons; blue: functional groups containing non-exchangeable protons. (**b**) NMR spectra of 100 mM mannitol (pH 6.5) in D_2_O (bottom, blue line) and H_2_O (top, red line, H_2_O: D_2_O = 90%:10%) at pH = 7.4 and room temperature. (**c**) Z-spectra and MTR_asym_ plots of 20 mM mannitol (PBS solution, pH 7.2, 37 °C) in PBS and blank PBS solution. CEST MRI was acquired using a CW pulse (B_1_ = 1.8 µT and T_sat_ = 4 s).

We first acquired and compared the NMR spectra of D-mannitol (100 mM, pH 6.5, room temperature) in H_2_O/D_2_O and D_2_O. Mannitol contains six exchangeable protons (red in Figure 1a) and eight non-exchangeable protons (blue in Figure 1a). As shown in Figure 1b, both exchangeable and non-exchangeable protons appeared in the H_2_O NMR spectrum, whereas only non-exchangeable protons were distinguishable in the D_2_O NMR spectrum. A broad signal of hydroxyl protons could be observed between 5.1 and 5.7 ppm, corresponding to offsets of 0.4 to 1.0 ppm with respect to the resonance of water (4.7 ppm). Four distinct NMR signals of non-exchangeable protons with distinct scalar coupling patterns were observed in both D_2_O and H_2_O NMR spectra, at approximately 3.65, 3.73, 3.77, and 3.84 ppm, in good agreement with previous reports [67]. Figure 1c shows a merged peak of water and OH signal in the Z-spectrum of 20 mM mannitol and a broad MTR_asym_ peak between 0.2 and 2 ppm. It is be (in the intermediate to fast exchange regime), leading to a distinct signal broadening especially at positive frequency and.

**Figure 2:**
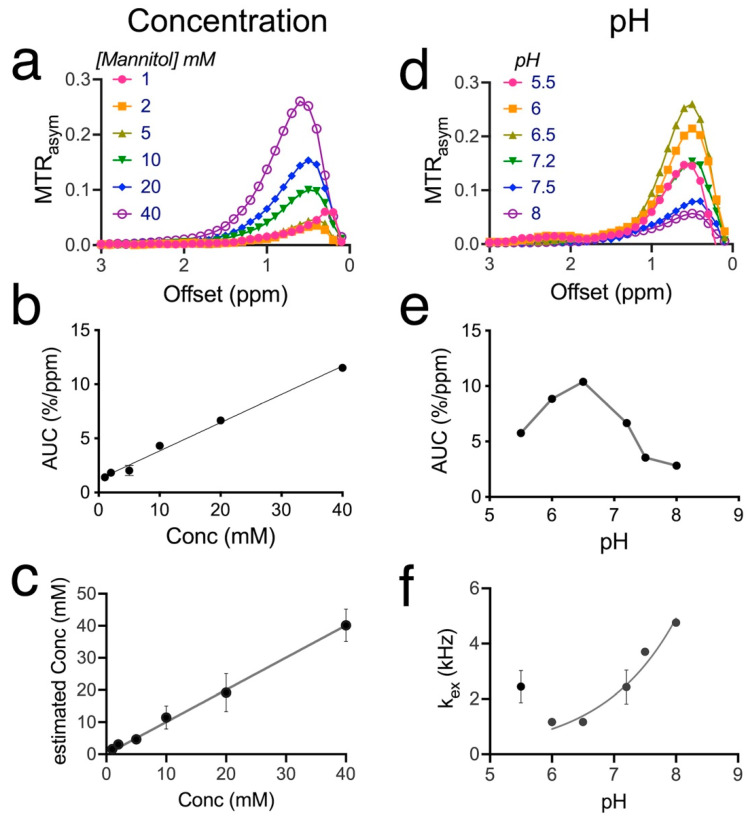
Concentration and pH dependence of the CEST MRI contrast of mannitol at 11.7T (37 °C). (**a**) MTR_asym_ plots of mannitol at concentrations ranging from 1 to 40 mM (pH 7.2 in PBS). (**b**) Concentration dependence of mannitol CEST contrast as quantified by AUC (0.2–2 ppm). (**c**) Linear correlation between the concentrations estimated using Bloch equation fitting and the nominal concentrations. (**d**) MTR_asym_ plots of mannitol solutions as a function of pH (20 mM in PBS). (**e**) pH dependence of mannitol CEST contrast as quantified by AUC (0.2–2 ppm). (**f**) Exchange rates of hydroxyl protons as a function of pH. Unless otherwise noted, all CEST experiments were acquired using a RARE sequence (TR/TE = 6000/5 ms) and a CW saturation pulse (B_1_/T_sat_ = 1.8 µT/4 s).

Subsequently, we characterized the CEST contrast of mannitol as a function of concentration and pH. As shown in Figure 2a, the CEST contrast of mannitol increases with concentration. The apparent peak position of the MTR_asym_ curve is around 0.4 ppm at 1 mM and shifts to higher offsets at higher concentrations, i.e., 0.6 ppm at 40 mM. Quantified by the AUC (0.2–2 ppm) of the MTR_asym_ plots, the mannitol CEST contrast is linearly correlated with concentration (Figure 2b, R^2^ = 0.9927). Fitting the Z-spectra to the Bloch equations allows more accurate estimation of the mannitol concentration for the faster exchange rates (Figure 2c, R^2^ = 0.9976). Based on the curves in Figure 2b,c, the detectability of CEST MRI for mannitol is estimated to be approximately 1 mM to generate 0.25 ± 0.01% per ppm CEST contrast (AUC (0.2–2 ppm)). Similar to D-glucose [34,35], the mannitol has CEST contrast that is sensitive to pH (Figure 2d,e) and decreases markedly with increasing pH for pH values above 6.5, attributed to the increased exchange rates and the faster exchange regime at higher pH. The CEST contrast at pH 6.5 was approximately 1.6 times higher than that at pH 7.2, with AUC values of 10.4 ± 0.2 and 16.7 ± 0.1% per ppm, respectively. The CEST contrasts at pH 5.5 and 6.0 (AUC = 5.8 ± 0.1% and 8.8 ± 0.2%/ppm, respectively) were smaller than that of pH 6.5, likely due to the shift from base-catalyzed to acid-catalyzed exchange regime. Using Bloch equation fitting of Z-spectra acquired at various B_1_ values (Figure S1), we estimated the exchange rates (k_ex_) of mannitol as a function of pH (Figure 2f), which showed that the exchange rate increases gradually between pH 6.5 t and 8.0. Even at pH 6.5, the k_ex_ was estimated to be 1167 s^−1^, which would still be considered intermediate, given the offset of 0.8 ppm (Δω = 0.8 ppm × 500 (s^−1^/ppm) × 2π (rad) ≈ 2500 rad/s).

The absolute CEST contrast is also impacted by the choice of acquisition parameters, including T_sat_ and B_1_, which were also studied (Figure S2). It should be noted that, while 3.6 µT appeared to be the optimal B_1_ for in vitro detection, we used a lower B_1_ value later in in vivo studies to reduce the MTC effect that may interfere with in vivo quantification. Finally, we also measured the r_1_ and r_2_ relaxivities of mannitol, which were 2.3 × 10^−5^ s^−1^ mM^−1^ and 5.7 × 10^−2^ s^−1^ mM^−1^ (pH = 7.2 and 37 °C), respectively (Figure S3), suggesting that mannitol causes a negligible T_1_ concentration effect and a noticeable T_2_ effect, similar to D-glucose [68].

### 3.2. Mannitol Excess Causes Extravasation and Brain Accumulation of Mannitol

**Figure 3:**
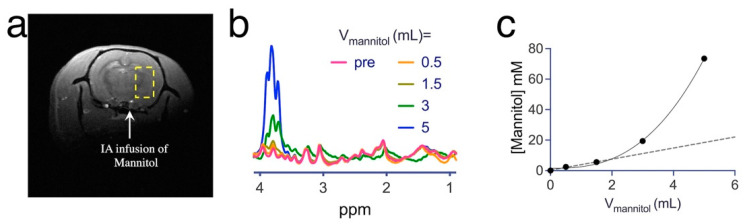
MRS assessment of the brain accumulation of administered mannitol (25% *w*/*w*). (**a**) Representative T_2w_ anatomical image showing the location of intra-arterial (IA) infusion (white arrow) of mannitol and the voxel (yellow dashed line) for ^1^H-MRS measurement. (**b**) ^1^H-MRS spectra as a function of the injection volume of mannitol. (**c**) Calculated mannitol concentration (mM) in the brain as a function of injection volume, where dash line is the linear regression of the first three data points of low doses.

We first investigated whether IA infusion of an excess amount of mannitol (at the ICA as illustrated in Figure 3a) could cause accumulation of mannitol in the injected hemisphere. We intermittently injected different volumes of 1374 mM (25% mannitol solution) intra-arterially and used ^1^H-MRS to detect brain uptake reflected in strongly increased aliphatic ^1^H signals in the range of 3.6–3.9 ppm (Figure 3b). Total mannitol concentration was estimated from the ratio of the integral of these aliphatic protons of mannitol (3.6–3.9 ppm, eight protons) with those of the integral total creatine at 3.03 ppm (assumed to be 9 mM [69], three protons). The mannitol concentrations in the brain were then calculated using a 0.34/11.7 mL blood to the total brain volume in the brain of rats [70] and a blood concentration of mannitol that was calculated using the injection volume (0.5 to 5 mL) and an average whole-body blood volume of 20.7 mL [70]. The quantitative MRS measures are shown in Figure 3c, which indicate a two-phase increase in the concentration of mannitol in the brain: an initially slow and linear increase phase in the injection volume range up to 2 mL, followed by a dramatic exponential increase phase when injection volume exceeded 2 mL. The leaked mannitol concentration reached a high level (>70 mM) when 5 mL mannitol was infused.

### 3.3. MRI Manifestations following Mannitol Accumulation in the Brain

**Figure 4:**
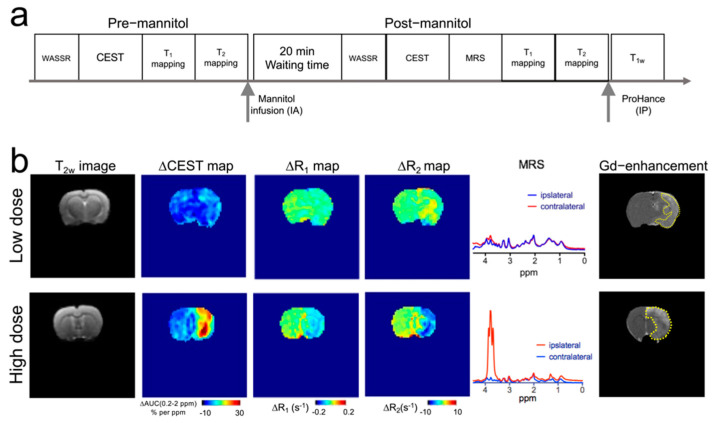
CEST, R_1_, R_2_, Gd-enhanced and MRS contrast changes caused by mannitol injected at low (1 mL) and high (3 mL) of 25% mannitol solution. (**a**) Schematic of the timeline of MRI acquisitions. (**b**) Representative images of the same slice showing (from left to right) T_2_-weighted anatomical images, CEST contrast parametric maps as quantified by ∆AUC (0.2–2 ppm), ∆R_1_ contrast parametric maps, ∆R_2_ contrast parametric maps; ^1^H MRS spectra, and Gd-based contrast-enhanced images of rats receiving low and high dose mannitol injection.

We subsequently conducted a comprehensive MR assessment, including CEST, T_1_, and T_2_ mapping, and ^1^H-MRS (Figure 4a), of rats receiving mannitol treatment at either low or high dose, i.e., injection volumes = 1 and 3 mL, respectively. The blood concentrations of mannitol were calculated to be 63 and 174 mM, respectively. At the end of each study, Gd-based contrast-enhanced MRI was also conducted to assess the area of BBB breakdown. Figure 4b shows the parametric maps of MRI contrast changes in representative rats from the two groups. It can be seen that IA infusion of mannitol at a low dose (1 mL) did not cause noticeable changes in CEST and T_1_ contrast. However, slightly increased R_2_ was observed in some of the regions.

Conversely, injecting an excessive amount of mannitol (3 mL) resulted in marked changes in CEST contrast, indicating the accumulation of mannitol at the injection site, which was confirmed by ^1^H-MRS observation. There were also markedly decreased R_1_ and R_2_ relaxation rates in the ipsilateral hemisphere, attributable to severe edema caused by the tissue accumulation of mannitol. Finally, the regions undergoing BBBO were rendered by Gd-based contrast-enhanced MRI, which revealed that, while both doses resulted in BBBO, the higher dose produced a much larger area of BBBO. In the high dose group, the BBBO regions appeared to be similar to those areas with increased mannitol-CEST contrast or those of decreased ∆R_1_, but larger than those of decreased ∆R_2_. In both groups, mannitol did not cause noticeable MRI contrast changes in the contralateral side, indicative of a negligible impact of mannitol infusion on the ”off-target” regions.

### 3.4. CEST MRI Detection of Mannitol in the Brain

**Figure 5:**
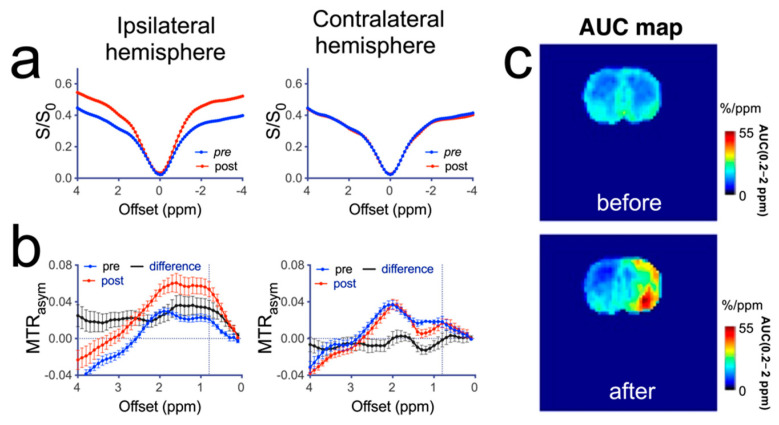
Quantitative analysis of CEST contrast in a representative rat infused with high dose (3 mL) of 25% mannitol solution. (**a**) Z-spectra obtained pre- and post-mannitol infusion in the ipsilateral (right) and contralateral (left) hemispheres. (**b**) MTR_asym_ plots of pre- and post-mannitol infusion in the ipsilateral (right) and contralateral (left) hemispheres. (**c**) AUC (0.2–2.0 ppm) maps before and after the infusion of mannitol.

**Figure 6:**
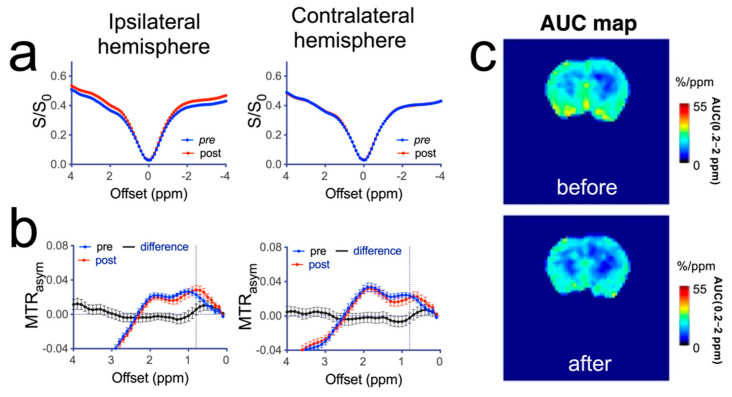
Quantitative analysis of CEST contrast in a representative rat infused with low dose (1 mL) of 25% mannitol solution. (**a**) Z-spectra of pre- and post-mannitol infusion in the ipsilateral (right) and contralateral (left) hemispheres. (**b**) MTR_asym_ plots of pre- and post-mannitol infusion in the ipsilateral (right) and contralateral (left) hemispheres. (**c**) AUC (0.2–2.0 ppm) maps before and after the infusion of mannitol.

We performed a quantitative analysis of the change in CEST contrast in different brain regions at about 20 min after mannitol infusion. As shown in Figure 5a, in rats that received high dose (3 mL) mannitol, there was a dramatic change in the mean Z-spectra of the ipsilateral hemisphere. No contrast change could be observed in the contralateral hemisphere. A main change in post-mannitol Z-spectra in the ipsilateral hemisphere was greatly reduced saturation transfer effect throughout the spectrum. On the contrary, MTR_asym_ analysis (Figure 5b) revealed a remarkable increase in CEST signal over the broad offset range corresponding to the mannitol hydroxyl signals. The AUC (0.2–2 ppm) parametric maps (Figure 5c) showed that the spatial distribution of increased CEST signal. The pre- and post-mannitol AUC (0.2–2 ppm) values (for the rat shown in Figure 5) were 20.1 ± 4.1%/ppm and 28.6 ± 5.1%/ppm in the ipsilateral hemisphere (*p* = 0.0433) and 20.0 ± 2.4%/ppm and 18.7 ± 2.6%/ppm in the contralateral hemisphere (*p* = 0.3787), respectively. In contrast, no significant change in CEST contrast was observed in the rats infused with 1 mL of 25% mannitol solution (Figure 6).

**Figure 7:**
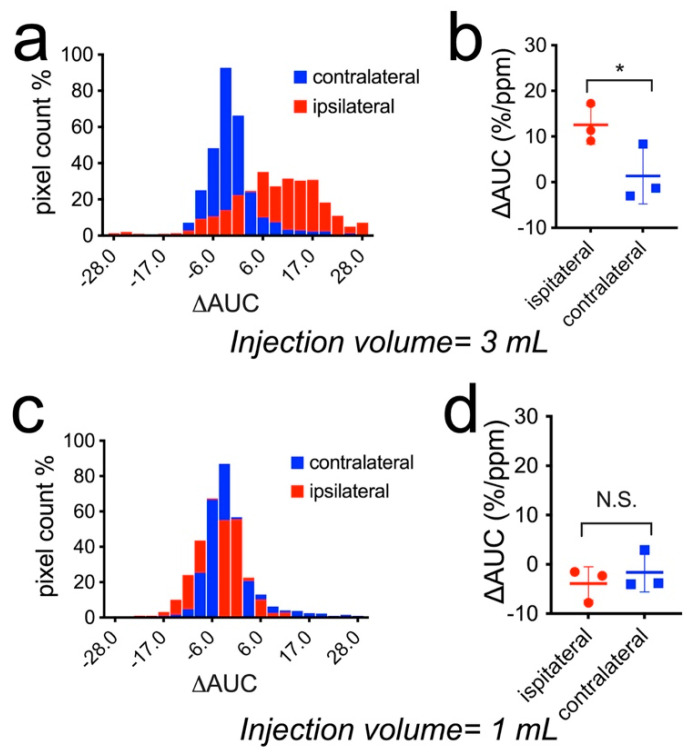
Comparison of the change in CEST contrast (∆AUC (0.2–2.0 ppm)) in the ipsilateral and contralateral hemispheres after the injection of different doses of mannitol. (**a**) Histogram and (**b**) Mean values of three rats injected with 3 mL 25% mannitol. *: *p* < 0.05 (*p* = 0.0105, paired two-tailed Student *t*-test, *n* = 3). (**c**) Histogram and (**d**) Mean values of three rats injected with 1 mL 25% mannitol. N.S.: not significant (*p* = 0.3536, paired two-tailed Student *t*-test, *n* = 3).

Figure 7 compares the mean CEST contrast changes in the two cohorts (n = 3 in each group). The histogram analysis shows that the CEST signals of the voxels in the high dose group were substantially increased, indicating mannitol accumulation. In addition, the mean ∆AUC (0.2–2.0 ppm) values were significantly higher in the ipsilateral hemisphere than contralaterally in rats that received 3 mL 25% mannitol (Figure 7b, 12.54 ± 4.23 vs. 1.34 ± 6.12%/ppm, *p* = 0.0105), while there was no significant difference the in low dose group (Figure 7d, −3.88 ± 3.40 vs. −1.63 ± 3.95%/ppm, *p* = 0.3536).

**Figure 8:**
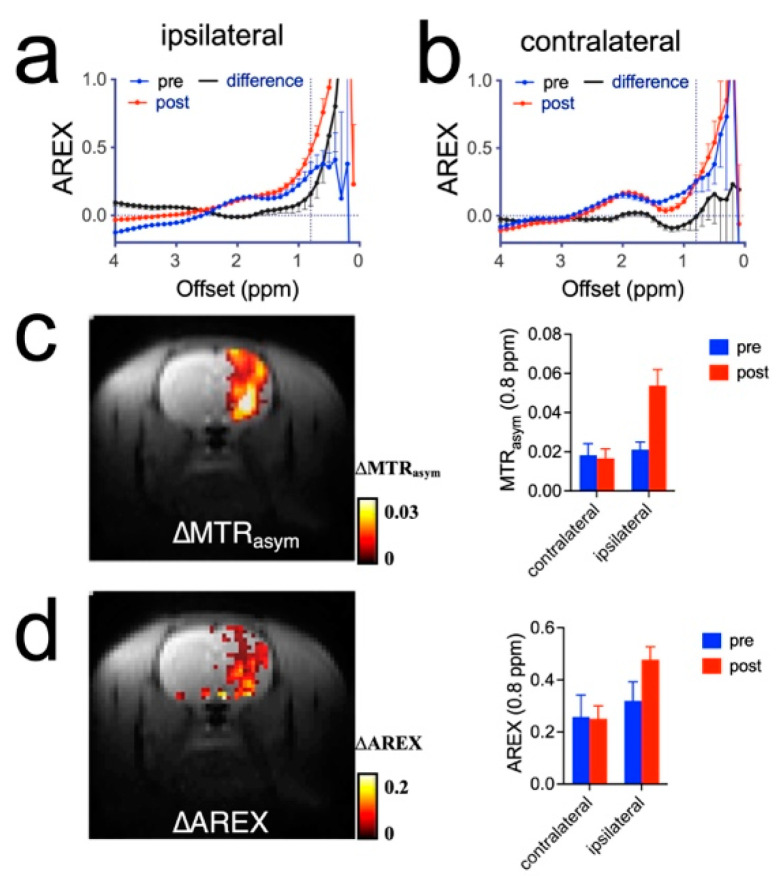
AREX analysis of rats infused with high dose (3 mL 25%) mannitol solution. (**a**) AREX plots of pre- and post-mannitol infusion in the ipsilateral hemisphere in a representative rat. (**b**) AREX plots of pre- and post-mannitol infusion in the contralateral hemisphere of the same rat. (**c**) Overlay image of T_2w_ image and ∆MTR_asym_ (0.8 ppm) parametric map (left) and the corresponding mean MTR_asym_ (0.8 ppm) in each hemisphere (right). (**d**) Overlay image of T_2w_ image and ∆AREX (0.8 ppm) parametric map (left) and the corresponding mean MTR_asym_ (0.8 ppm) in each hemisphere (right).

To accurately quantify the CEST effect in the presence of substantial T_1_ changes, we also attempted to quantify the CEST contrast enhancement by mannitol using the AREX approach. Figure 8a,b show the AREX (or AREX_asym_) plots in the ipsilateral and contralateral hemispheres in a rat receiving high dose mannitol. Comparing the ∆MTR_asym_ (0.8 ppm) maps (Figure 8c) and ∆AREX (0.8 ppm) map (Figure 8d) reveals good agreement between the spatial distribution of ∆CEST signal by the two methods although the CEST contrast enhancement calculated by the MTR_asym_ method (i.e., 0.021 ± 0.003 vs. 0.054 ± 0.008 for pre- and post-mannitol, respectively, corresponding to an increase of 157% in CEST contrast) was higher than that by the AREX method (i.e., 0.318 ± 0.07 vs. 0.477 ± 0.05 for pre- and post-mannitol, respectively, corresponding to an increase of 50% in CEST contrast), attributed to T_1_ “contamination” in the MTR_asym_ method.

**Figure 9:**
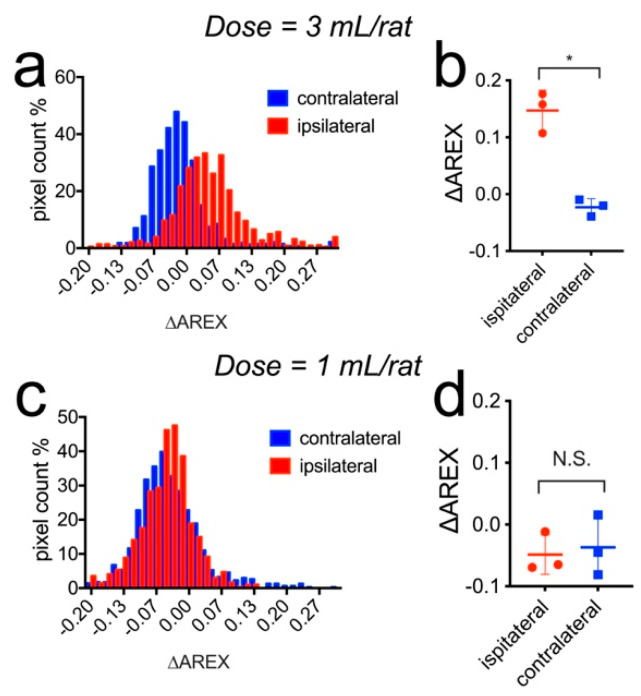
Comparison of the change in CEST contrast (∆AREX) in the ipsilateral and contralateral hemispheres after the injection of mannitol at different doses. (**a**) Histogram and (**b**) Mean values of three rats injected with 3 mL 25% mannitol. *: *p* = 0.0237, paired two-tailed Student *t*-test, n = 3. (**c**) Histogram and (**d**) Mean values of three rats injected with 1 mL 25% mannitol. N.S.: *p* = 0.4297, paired two-tailed Student *t*-test, *n* = 3.

The comparison of the mean ∆AREX values between the low and high dose groups (n = 3 each) is shown in Figure 9. The histogram analysis shows that the CEST signals of the voxels in the high dose group were substantially inclined, indicating mannitol accumulation. In addition, the mean ∆AREX values were significantly higher in the ipsilateral hemispheres than those in the contralateral hemisphere in rats that received 3 mL 25% mannitol (Figure 9b, 0.147 ± 0.036 vs. −0.023 ± 0.015, *p* = 0.0237), while there was no significant difference the in low dose group (Figure 9d, −0.049 ± 0.032 vs. −0.037 ± 0.049, *p* = 0.4297).

Finally, we also attempted to quantify the CEST effect using Bloch fitting of the acquired CEST Z-spectral data together with T_1_ and T_2_ data (Figure S4) to compute the average concentration of mannitol in the two hemispheres. The average ipsilateral mannitol concentration in the high dose group (*n* = 3) was estimated to be 23.8 ± 17.8 mM, which is in good agreement with that measured by the MRS method (24.1 ± 4.9 mM), whereas the average concentration of mannitol was estimated to be 4.5 ± 3.7 mM in the low dose group (*n* = 3).

## 4. Discussion

In the present study, we systematically characterized the inherent CEST MRI contrast of mannitol *in vitro* and exploited it for non-invasive and label-free detection of mannitol accumulated in the brain following intra-arterial infusion for OBBBO. While mannitol has been used as a hyperosmotic agent for various medical applications, including OBBBO, it can cause severe adverse events in some patients (especially those with renal dysfunction), including electrolyte abnormalities, acidosis, hypotension, acute renal failure congestive, and heart failure with pulmonary edema [71,72,73,74]. Mannitol has been widely used to reduce ICP in the context of progressive symptomatic brain edema. Mounting evidence shows the correlation between excessive use of mannitol and mannitol accumulation in the areas of trauma, infarction, and tumors, where the BBB is disrupted [24,25,26]. Recently, there has been a renewed interest in using mannitol as a hyperosmotic agent to selectively open BBB near lesions in the central nervous system (CNS) through intra-arterial catheterization [75]. It was reported that a single injection of mannitol could result in an effective transient BBBO for 30 to 60 min, which returned to baseline completely in 2 to 10 h [76]. Catheter position, infusion speed, and infusion volume collectively determine the spatial distribution and extent of BBBO [16,17,18]. In an OBBBO treatment, if injected at an excessive amount, mannitol tends to leak into the region via the opened BBB. Our study concurred with this knowledge and showed as high as 70 mM mannitol could leaked to the brain parenchyma. Brain accumulation of mannitol was shown to be associated with consequent increase in water content or edema (indicated by increased regional T_1_ and T_2_ times). These findings are consistent with previously reported mannitol accumulation in the acute cerebral ischemia [30] or brain tumors and surrounding peritumoral edematous parenchyma [28,29]. In contrast, when the infusion volume was relatively small (i.e., 1 mL of 25% mannitol or less), the leakage of mannitol into the interstitial space was found insignificant, correlating well with CEST MRI observation. Yet this dose was still sufficient to open the BBB as evidenced by gadolinium contrast. Therefore, CEST MRI provides a useful non-invasive imaging tool to optimize a mannitol-based BBBO treatment in which efficacy and adverse effects are properly balanced. Of note, the infusion time is also a critical factor, and extended infusion time can lead to thrombus formation and thereby ischemia. In fact, our MRS result (Figure 4b) revealed the augmented production and accumulation of lactate (1.3 ppm) in the ipsilateral hemisphere in some rats receiving a high dose (3 mL) using a long infusion time (5 min), indicative of ischemia.

CEST MRI provides an effective non-invasive imaging tool for specifically assessing the amount of mannitol accumulated in tissues, which is of clinical significance. The methods currently being used in the clinic, including serum osmolarity or osmolar gap, are invasive and indirect. Only a few electrochemical-sensors-based [77] or enzyme-based (e.g., mannitol dehydrogenase) [24] ex vivo methods have been developed to date, but none of them enters clinical applications yet. Only few mannitol-detecting MRI methods have been reported previously. For instance, chemical shift imaging has been demonstrated to detect the spatial distribution of mannitol in acute cerebral ischemia [30] and meningioma [29]. However, low spatial and temporal resolution hinders the widespread clinical use of the ^1^H-MRS/CSI method. Compared to ^1^H MRS, CEST MRI has relatively higher sensitivity, higher spatial resolution, and shorter acquisition time; hence it is more suitable for monitoring mannitol distribution in vivo in a clinical setting. Spin-lock (CESL) MRI has also been used to detect D-mannitol [36,78]. CEST and CESL MRI are inherently very similar, with both methods detecting exchangeable protons. In the CESL studies by Jin [36,78], mannitol was used as an osmolality control to investigate the contribution of altered tissue water content to the apparent ∆R1*ρ*. Being used as an intravascular agent in those studies, no quantitative detection of tissue mannitol was performed. Here, we applied CEST MRI for detecting mannitol accumulated in the brain. Our study is the first attempt to perform quantitative analyses of tissue-accumulated mannitol using label-free CEST MRI directly by its hydroxyl protons. Further technical optimization is warranted to improve the accuracy of quantification. For example, we observed relatively large variations using a 6-pool Bloch fitting, likely due to low SNR of pixel-wise CEST data. We then demonstrated that AREX (also called AREX_asym_) was helpful in reducing the influence of T_1_ and T_2_. Still, future studies are needed to convert the measured AREX values into mannitol concentrations and validate such quantitation. Another technical hurdle is the potentially low sensitivity of CEST MRI detection at low field strengths, such as 3T. For example, the simulation (Figure S5) showed that the sensitivity may be as much as three times lower at 3T than at 9.4T. While previous studies on glucose-enhanced CEST MRI were successful at 3T scanners, both preclinically [79] and clinically [49,80], it will require a higher concentration to generate sufficiently high CEST MRI at 3T. One possible solution is to use advanced CEST MRI methods, such as CESL and on-resonance variable delay multipulse (VDMP), which may be more suitable for detecting fast exchangeable protons, such as hydroxyl protons, at 3T [48,78]. 

Lastly, the present approach could be particularly useful when OBBBO is combined with the delivery of chemotherapy agents that can be detected by MRI based on their inherent CEST signatures, such as gemcitabine [81,82], pemetrexed [83], olsalazine [84], and melphalan [85], just to name a few. The combined CEST could concurrently optimize mannitol dosing, the timing and dosing of the anticancer agent to be delivered and assess whether therapeutically desirable drug levels are achieved within the target volume. Generating such data is not only crucial for individualized medicine, but was also recently recommended as one of the baseline requirements for novel agents to be considered for phase II/III brain cancer efficacy trials [2].

It should be noted that accurate determination of the concentration of mannitol using CEST MRI is not always straightforward because the CEST MRI signal is affected by a wide variety of factors. For example, T_1_ is known to strongly affect CEST quantification. In our study, we found the tissue T_1_ and T_2_ relaxation times were markedly increased in the high dose group, attributed to edema formation. In this context, T_1_-compensated methods such as the T_1_ compensated inverse Z-analysis (AREX) can be used to reduce the potential influence of tissue T_1_ changes [59,60,61]. Our results showed that in the regions with strong T_1_ changes, the degree of ∆AREX increase was much smaller than that of ∆MTR_asym_, indicative of an overestimation of mannitol accumulation when the non-T_1_-compensated MTR_asym_ was used. However, both methods show consistent spatial distribution of mannitol (Figure 8). The AREX-based analyses also showed significantly elevated CEST in the ipsilateral hemispheres compared to the contralateral hemisphere in the high mannitol dosing group, suggesting the observed CEST signal change was mainly the result of mannitol accumulation than tissue T_1_ changes. Moreover, we utilized Bloch equation fitting to extract mannitol concentration in the tissue. This method takes all factors, such as T_1_, T_2_, tissue water content, and other exchangeable protons, into consideration and hence potentially provides more accurate quantification of mannitol concentration. Indeed, the computed mannitol concentrations were in good agreement with MRS measurement. Tissue pH might also affect the accuracy of quantification. While both intracellular and extracellular pH values in the healthy brain are constant [24], pathological changes such as acute ischemia can cause tissue pH to vary significantly [86]. Hence, caution has to be taken to quantify the mannitol concentration in different compartments. Injecting a relatively large volume of mannitol solution may also cause a transient change in the blood pH (normal range ~7.4) because the solution has a relatively acidic pH of 5.9 (4.5 to 7.0). However, the CEST MRI detection was performed 20 min later; hence, this transient pH change is unlikely to impact our CEST MRI measurement significantly. All those factors should be considered when converting the measured CEST signal to mannitol concentration. Given that the NMR signals of non-exchangeable protons acquired by ^1^H-MRS are not prone to these factors, combining CEST and MRS can achieve both quantification accuracy and high spatial/temporal resolution for detecting mannitol.

## 5. Conclusions

We characterized the CEST MRI contrast of mannitol and demonstrated that CEST MRI could be used for non-invasive, label-free detection of mannitol accumulated in the brain parenchyma following OBBBO. Thus, the present CEST MRI method provides a tool for monitoring the brain uptake of mannitol and adjusting the dose in mannitol-based treatments to obtain optimized efficacy and safety. Furthermore, there is traction to move guidance of IA infusions to cerebral vasculature from the current X-ray routine toward real-time MRI guidance. In this new environment, the reported MRI method has potential for improving the safety profile of OBBBO by continuously monitoring mannitol accumulation in the brain. It could be combined with previously described, drug-detecting CEST techniques to confirm adequate drug delivery after OBBBO.

## Figures and Tables

**Table 1:** List of the starting or fixed values of the parameters used in Bloch fitting.

	Hydroxyl	Amine/Guanidinium	Amide	NOE	MTC
Proton concentration (mM)	45	100	72	100	5500
Exchange rate (Hz)	1400	1100	30	16	15
Offset (ppm)	0.9	***2***	***3.5***	***−3.5***	***−2.3***
T2 (ms)	***55***	***170***	***100***	**5**	***9.1 × 10^−3^***
T1 (s)	***1***	***1***	***1***	***1***	***1***

Italic/bold numbers indicate those values were fixed in the fitting.

## Data Availability

Data presented in this study are available on request from the corresponding author.

## Supplementary Materials

The following supporting information can be downloaded at: https://www.mdpi.com/article/10.3390/pharmaceutics14112529/s1, References [87,88,89,90,91,92] are cited in the supplementary materials. Figure S1. Bloch fitting (lines) of the experiment data (solid dots) of 20 mM mannitol at pH 7.2 (37 °C, PBS). Figure S2. Optimization of CEST MRI detection of mannitol. Figure S3. R_1_ and R_2_ relaxation rates of mannitol at different concentrations at pH 7.2 (37 °C, PBS). Figure S4. Estimation of the change in hydroxyl proton concentration due to mannitol accumulation in the brain by Bloch fitting of Z-spectral data. Figure S5. Comparison of the CEST signal at 3T and 9.4T.
