# Supplementary material for: Label-Free Assessment of Mannitol Accumulation Following Osmotic Blood–Brain Barrier Opening Using Chemical Exchange Saturation Transfer Magnetic Resonance Imaging
Source: Pharmaceutics. 2022 Nov 20;14(11):2529. doi: 10.3390/pharmaceutics14112529 (PMC9695341; doi:10.3390/pharmaceutics14112529)
Supplement: Supplementary file 1 [file pharmaceutics-14-02529-s001.zip › pharmaceutics-2005916-supplementary.pdf]

Supporting information

Label-Free Assessment of Mannitol Accumulation following Osmotic Blood-Brain Barrier Opening Using Chemical Exchange Saturation Transfer Magnetic Resonance Imaging

Jing Liu[1,2,3 †], Chengyan Chu[4†], Jia Zhang[2,3], Chongxue Bie[2,3], Lin Chen[2,3], Safiya Aafreen[5], Jiadi Xu[2,3], David O. Kamson[6,7], Peter C. M. van Zijl[2,3], Piotr Walczak[4], Miroslaw Janowski[4], and Guanshu Liu[2,3,6] *

1. Department of Radiology, The First Affiliated Hospital Of Guangzhou Medical University, Guangzhou 510230, Guangdong
2. Russell H. Morgan Department of Radiology and Radiological Sciences, Division of MR Research, The Johns Hopkins University School of Medicine, Baltimore, MD 21205, USA
3. F.M. Kirby Research Center for Functional Brain Imaging, Kennedy Krieger Institute, Baltimore, MD 21205, USA
4. Department of Diagnostic Radiology and Nuclear Medicine, University of Maryland, Baltimore, Maryland, USA
5. Department of Biomedical Engineering, Johns Hopkins University, Baltimore, MD 21201, USA
6. The Sidney Kimmel Comprehensive Cancer Center at Johns Hopkins University, Baltimore, MD 21218, USA
7. Department of Neurology, Johns Hopkins University, Baltimore, MD 21218, USA

† These authors contributed equally to this work.
* Corresponding author:
Guanshu Liu
707 N. Broadway, Baltimore, MD 21205
Phone (office): +1-443-923-9500; Fax: +1-410-614-3147
Email: guanshu@mri.jhu.edu

S1. Estimation of exchange rates using Bloch fitting of Z-spectra data at various B_1 field strengths.

Two-pool Bloch equation fitting was used to simultaneously fit Z-spectra acquired at five B_1 field strengths according to previously published protocols [1-3]. Fitting was conducted using the open source Matlab scripts downloaded from cest-sources.org (https://www.cest-sources.org/doku.php?id=multi-b1-fit-cw) that were developed by Zaiss et al [4]. The relative proton concentration was f_b = 20 (mM) x6 (protons) /111 (M) = 1.1 x10^{-3}. The water-pool relaxation rates in phantoms were set to R_{1A} = 0.217 s^{-1} and R_{2B} = 0.454 s^{-1}, and relaxation rates of the CEST pool were set fixed to R_{1B} = 1 s^{-1}, R_{2B} = 50 s^{-1}. Other fixed parameters included T_{sat} = 4 sec, recovering time T_{rec} = 2 sec; $\Delta\omega$ = 0.8 ppm.

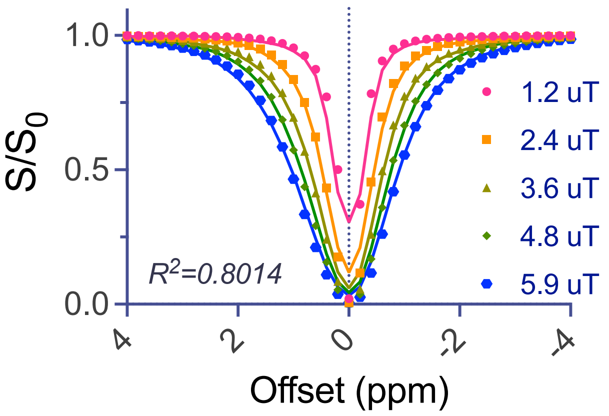

Figure S1. Bloch fitting (lines) of the experiment data (solid dots) of 20 mM mannitol at pH 7.2 (37˚C, PBS).

S2. Optimization of CEST acquisition parameters

The relationship between CEST contrast and CEST acquisition parameters was investigated, i.e., B_1 and T_{sat}. As shown in **Figures S2a** and **S2c**, the peak position of the MTR_{asym} plots of mannitol shifted with both parameters, suggesting that the quantification of CEST MRI using the MTR_{asym} values at 0.8 ppm might lead to errors due to interference of direct water saturation. For simplicity, we decided to use AUC (0.2 -2 ppm) values calculated from the MTR_{asym} curves to quantify CEST contrast at different conditions. **Figure S2b** shows that the CEST contrast of mannitol leveled off between 2 - 3 sec, indicating that 3 sec can be considered an optimal T_{sat} value. The B_1 curve (Figure S2d) shows that 3.6 μT can be approximately considered as the optimal B_1 value at T_{sat} = 3 s in phantoms. However, we used a lower B_1 value (1.8 μT) in our *in vivo* studies because high B_1 results in a pronounced magnetic transfer (MT) contrast that may further complicate the CEST detection and quantification.

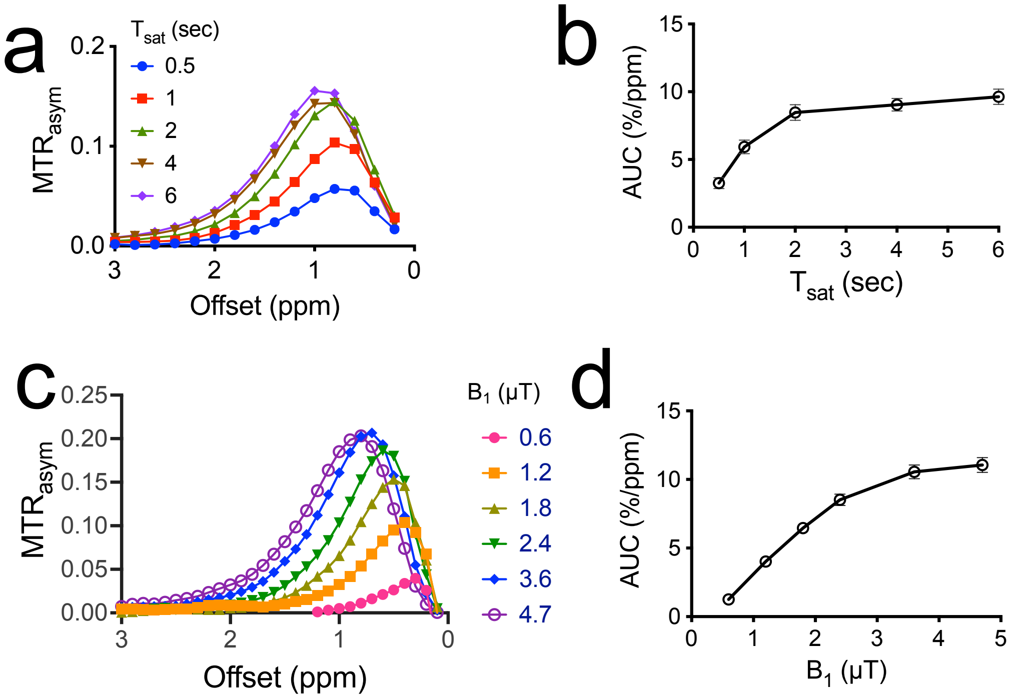

Figure S2. Optimization of CEST MRI detection of mannitol. **a**) MTR_{asym} plots and **b**) AUC (0.2-2 ppm) of 20 mM mannitol (pH 7.4, 37 °C) as a function of T_{sat}. CEST MRI was acquired using a RARE sequence (TR/TE= 10,000/5 ms) and a CW saturation pulse (B_1 = 1.8 μT). **c**) MTR_{asym} plots and **d**) AUC (0.2-4 ppm) of 20 mM mannitol (pH 7.4, 37 °C) at different B_1 values. CEST MRI was acquired using a RARE sequence (TR/TE= 6000/5 ms) and a CW saturation pulse (T_{sat}=3 sec) at 37 °C.

S3. T_1 and T_2 effects of mannitol

T_1 and T_2 maps were acquired according to our previously published procedures [5]. In brief, T_1 maps were acquired using a RARE-based saturation recovery sequence with eight TR values ranging between 200 ms to 15,000 ms (TE = 4.3 ms and RARE factor = 4, central encoding). T_2 maps were acquired using a modified RARE pulse sequence (TR/TE = 25000/4.3 ms and RARE factor = 8) with a Carr-Purcell-Meiboom-Gill (CPMG) T_2 preparation module consisting of a CPMG pulse train (t_{CPMG} = 10 ms). A total of 16 scans were acquired with the number of CPMG loops varied from 2 to 1024, corresponding to TE times of 20 ms to 10.24 sec.

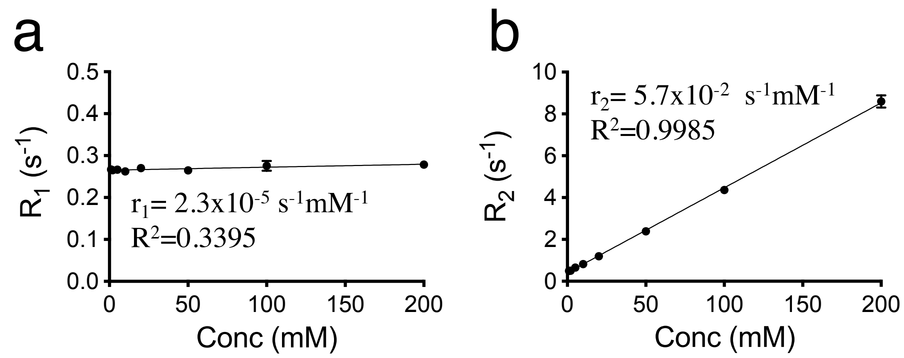

Figure S3. R_1 (**a**) and R_2 (**b**) relaxation rates of mannitol at different concentrations at pH 7.2 (37˚C, PBS).

S4. In vivo quantification of mannitol concentration using Bloch fitting of Z-spectral data

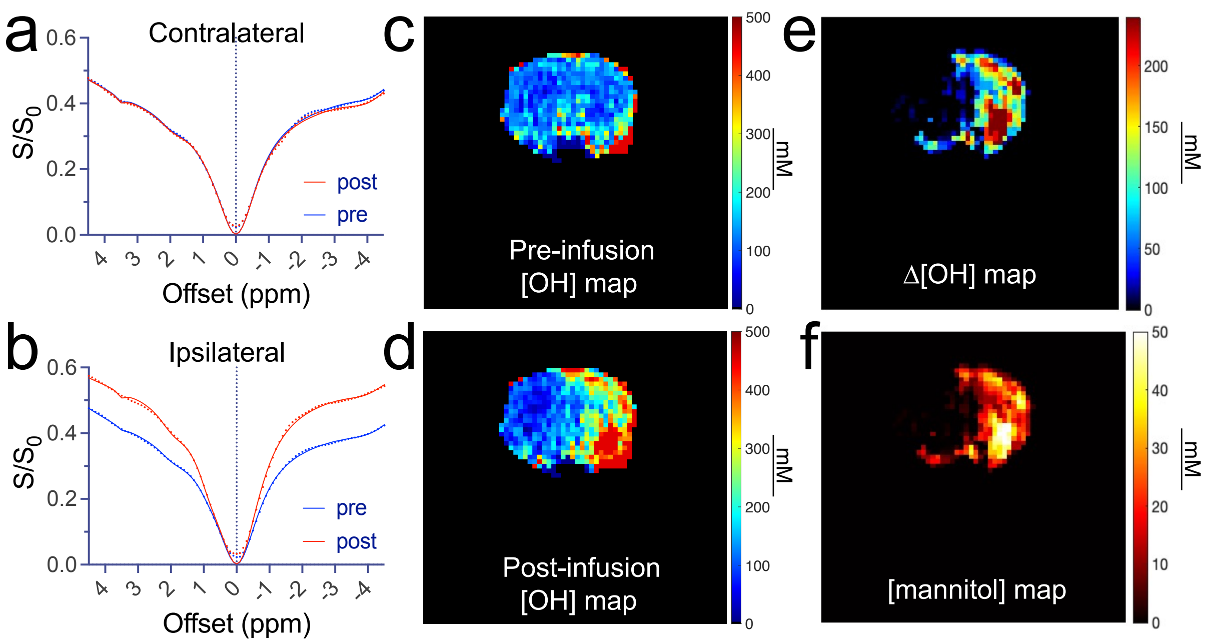

Figure S4. Estimation of the change in hydroxyl proton concentration due to mannitol accumulation in the brain by Bloch fitting of Z-spectral data. (a) Experimental and fitted Z-spectra of the contralateral hemisphere pre and post mannitol infusion. **(b)** Experimental and fitted Z-spectra of the ipsilateral hemisphere pre and post mannitol infusion. Parametric maps of the concentration of Hydroxyl protons (mM) in a representative rat pre- **(c)** and post- **(d)** infusion of 3 mL mannitol. (**e**) Parametric map showing the change in hydroxyl proton concentration. (**f**) Computed concentration map of mannitol.

S5. Simulation of the mannitol CEST contrast at 3T and 9.4 T.

To estimate the CEST contrast of mannitol at 3T, we performed simulations using 2-pool Bloch McConnel equations realized using MATLAB (Mathworks, Natick, MA). The following parameters were used in the simulation, a continuous wave (CW) pulse with a duration of 4 seconds and a B1 field strength of $\omega 1/2\pi$ = 1.8 μT, the chemical shift =0.8 ppm, and k_{ex} = 2.4 kHz. Two field strengths, 3 and 9.4 Tesla, were used. Of note, 9.4 T was chosen because the availability of literature-reported values of relaxation times in the brain. We used the literature reported water relaxation times of white Matter (WM): T_{1W} =1.66 sec and T_{2W} =37.2 ms at 9.4 T [6]; T_{1W} =1.10 sec and T_{2W} =69 ms at 3T [7]. For hydroxyl protons, the T_1 time was the same as that of water and the T_2 was 94 ms respectively [8]. The concentration of mannitol was set to 50 mM. The result shows that the peak value of mannitol CEST is approximately four times higher at 9.4T than at 3T.

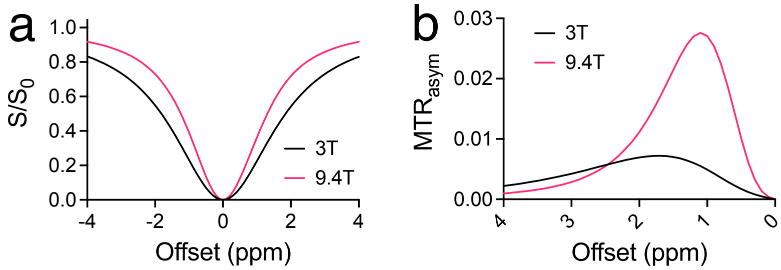

Figure S5. Comparison of the CEST signal at 3T and 9.4T. a) Z-spectra. **b)** MTR_{asym} plots.

References

1 Randtke, E.A.; Chen, L.Q.; Pagel, M.D. The reciprocal linear QUEST analysis method facilitates the measurements of chemical exchange rates with CEST MRI. *Contrast Media Mol Imaging* **2014**, *9*, 252-258, doi:10.1002/cmmi.1566.

2 Zaiss, M.; Anemone, A.; Goerke, S.; Longo, D.L.; Herz, K.; Pohmann, R.; Aime, S.; Rivlin, M.; Navon, G.; Golay, X.; et al. Quantification of hydroxyl exchange of D-Glucose at physiological conditions for optimization of glucoCEST MRI at 3, 7 and 9.4 Tesla. *NMR Biomed.* **2019**, *32*, e4113, doi:10.1002/nbm.4113.

3 Zaiss, M.; Angelovski, G.; Demetriou, E.; McMahon, M.T.; Golay, X.; Scheffler, K. QUESP and QUEST revisited—Fast and accurate quantitative CEST experiments. *Magn. Reson. Med.* **2018**, *79*, 1708-1721, doi:10.1002/mrm.2681.

4 Zaiss, M. CEST Sources. 2014. Availabe online: http://www.cest-sources.org (accessed on 1 Feburary 2022).

5 Zhang, J.; Li, Y.; Slania, S.; Yadav, N.N.; Liu, J.; Wang, R.; Zhang, J.; Pomper, M.G.; van Zijl, P.C.; Yang, X.; et al. Phenols as Diamagnetic T2 -Exchange Magnetic Resonance Imaging Contrast Agents. *Chemistry (Easton)* **2018**, *24*, 1259–1263.

6 de Graaf, R.A.; Brown, P.B.; McIntyre, S.; Nixon, T.W.; Behar, K.L.; Rothman, D.L. High magnetic field water and metabolite proton T1 and T2 relaxation in rat brain in vivo. *Magn. Reson. Med.* **2006**, *56*, 386-394, doi:10.1002/mrm.20946.
7 Stanisz, G.J.; Odrobina, E.E.; Pun, J.; Escaravage, M.; Graham, S.J.; Bronskill, M.J.; Henkelman, R.M. T1, T2 relaxation and magnetization transfer in tissue at 3T. *Magn. Reson. Med.* **2005**, *54*, 507-512, doi:10.1002/mrm.20605.
8 Hills, B.; Cano, C.; Belton, P. Proton NMR relaxation studies of aqueous polysaccharide systems. *Macromolecules* **1991**, *24*, 2944-2950.
